# Brain Activation for Social Cognition and Emotion Processing Tasks in Borderline Personality Disorder: A Meta-Analysis of Neuroimaging Studies

**DOI:** 10.3390/brainsci14040395

**Published:** 2024-04-18

**Authors:** Matthias Schurz, Jan-Patrick Berenz, Jeff Maerz, Raphael Perla, Anna Buchheim, Karin Labek

**Affiliations:** 1 Department of Psychology, Faculty of Psychology and Sport Science, and Digital Science Center (DiSC), University of Innsbruck, Universitätsstrasse 15, 6020 Innsbruck, Austria; 2 Department of Psychology, Faculty of Psychology and Sport Science, University of Innsbruck, Universitätsstrasse 15, 6020 Innsbruck, Austria

**Keywords:** fMRI, neuroimaging, meta-analysis, borderline personality disorder, social cognition, mentalizing, empathy, emotion processing, self–other distinction

## Abstract

The present meta-analysis summarizes brain activation for social cognition and emotion-processing tasks in borderline personality disorder (BPD). We carried out two meta-analyses to elaborate on commonalities and potential differences between the two types of tasks. In the first meta-analysis, we implemented a more liberal strategy for task selection (including social and emotional content). The results confirmed previously reported hyperactivations in patients with BPD in the bilateral amygdala and prefrontal cortex and hypoactivations in bilateral inferior frontal gyri. When applying a stricter approach to task selection, focusing narrowly on social cognition tasks, we only found activation in prefrontal areas, particularly in the anterior cingulate and ventromedial prefrontal cortex. We review the role of these areas in social cognition in healthy adults, suggesting that the observed BPD hyperactivations may reflect an overreliance on self-related thought in social cognition.

## 1. Introduction

Borderline personality disorder (BPD) is a severe mental health condition with a prevalence of around 10%, which negatively affects multiple areas of life [1,2,3]. Patients typically show symptoms concerning their emotionality and social interactions: variable affect and pronounced impulsivity, emotional dysregulation, unstable patterns of interpersonal relationships and self-image [4]. Emotional disturbances have been one topical focus (e.g., [5,6,7]) in BPD research. Based on mounting evidence on the subject, literature reviews and meta-analyses [6,7,8,9,10] have broadly linked aberrant emotion processing to two neural abnormalities: the hyperactivation of the amygdalae, reflecting excessive emotional responding, and hypoactivation in prefrontal areas linked to impaired emotion regulation. Recently, two large-scale clinical meta-analyses [11,12] found that these aberrant patterns of emotion processing and cognitive control in the brain are a common characteristic of multiple psychiatric disorders, including, for example, depression, anxiety, substance use disorder, and schizophrenia. In addition to disturbed emotion, outstanding characteristics of borderline personality are problems that manifest in social life and interpersonal relationships (e.g., [1,2,13,14,15,16,17,18]). In clinical and behavioral assessments, patients with BPD show impairments in the ability to correctly infer the mental states of other people (for review, see [19,20,21]; for psychotherapy studies, see [22,23]). It has been suggested [21,24,25,26] that patients with BPD strongly rely on fast and automatic forms of mentalizing in combination with problems in controlled and cognitive mentalizing. However, the study of brain abnormalities linked to social cognition and mentalizing has received comparably little attention in meta-analyses of borderline personality disorder.

Concerning the neural correlates of social cognition in healthy adults, meta-analyses have produced an increasingly detailed picture of evidence over the past decade, thanks to the continuing increase in published studies (see [27,28,29,30,31,32,33]). In the present study, we seek to build on these insights by selecting studies on borderline personality disorder that involve social processes. In particular, we build upon a model of social cognition, which we developed in a previous meta-analysis [32]. In this systematic literature synthesis, we clarified the interrelations between diverse forms of social cognition in terms of their neural basis. We carried out a meta-analysis of two central forms of social cognitive processes. The first form was empathy, generally referring to an affective route for making sense of others (e.g., [34,35]). The second form was mentalizing, also referred to as theory of mind (ToM), which denotes understanding others’ mental states via more cognitive processes (e.g., [36,37,38,39]). By clustering brain activation maps for different tasks measuring the two different social abilities, we could show that a considerable amount of processes engaged by empathy and mentalizing rely on common brain networks. In the second step, we reviewed other meta-analyses and empirical studies on related forms of social cognition. The inspection of maps showed multiple overlaps among processes, which could be summarized by two overarching networks linked to more sensory–affective versus more abstract and decoupled representations of others’ mental states. We also observed that several forms of social cognition recruited the sensory–affective and cognitive–abstract networks conjointly. Together, the two identified brain networks featured component processes not only implicated in empathy and mentalizing (see [32], p. 20) but also in action observation [40], emotion processing [41], experiencing social exclusion [42], and social interactions (e.g., [43]; see also [32], p. 23).

The present meta-analysis aims to further specify brain abnormalities for processing emotional and social stimuli in borderline personality disorder by selecting studies based on tasks and stimuli that connect with our integrative model of social cognition [32]. Our study contributes new meta-analytic evidence on the neural correlates for social cognition in borderline personality disorder, a question receiving limited attention in existing meta-analyses [8,9,10].

## 2. Materials and Methods

### 2.1. Literature Search

**Figure 1:**
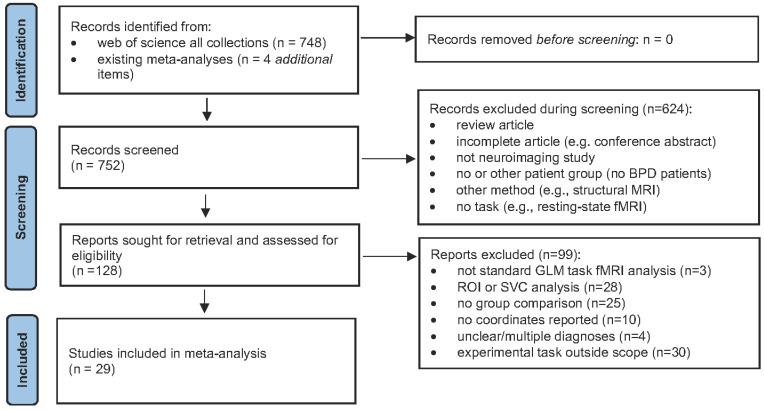
The PRISMA flow diagram, describing the literature selection process. We identified 748 items from a database search in the Web of Science platform (all collections). After the abstract screening, 128 manuscripts were assessed for eligibility. From those, we identified 29 studies for our meta-analysis on social cognition in borderline personality disorder.

Our method follows the guidelines for a neuroimaging meta-analysis with SDM-PSI [44] and additional recommendations from the PRISMA guidelines [45]. We searched for eligible studies on all databases and all collections of the Web of Science platform (www.webofscience.com (accessed on 14 April 2024)), with a search date of 31 January 2023. To identify neuroimaging studies, we used the keyword combination “functional magnetic resonance imaging” or “positron emission tomography” or (“functional” and “magnetic” and “resonance” and “imaging”). To specify the clinical topic of our meta-analysis, we used the keyword combination (“borderline” and “personality”) or “borderline personality disorder” or “bpd”. We excluded documents of the type “review article” from the list of retrieved documents, resulting in 748 identified items. In addition, we studied the samples of recently published neuroimaging meta-analyses on BPD [9,10] and added all items that were not already on our list. After screening abstracts, 128 studies were retained, and the corresponding full-text manuscripts were assessed for eligibility (see Figure 1). Studies had to fulfill standard selection criteria for a meta-analysis (see [46]). These criteria were assessed independently by the first and second authors (M.S. and J.-P.B.) and, if necessary, reviewed and discussed until those authors reached an agreement. Concretely, manuscripts had to report results from a task-based neuroimaging (fMRI or PET) study, analyzed with a GLM approach at the whole-brain level, which used a consistent statistical threshold throughout the brain. This criterion excludes studies using Region of Interest (ROI) or small-volume correction approaches. Reported coordinates had to conform to standard space (MNI or TAL). If a study reported brain activation for multiple contrasts, we prioritized comparisons against a well-matched control condition (not baseline) and the comparisons/conditions that best corresponded to the other contrasts in the sample. To achieve a sufficiently large sample of studies, we also included eight studies that did not use a well-matched control condition (see Table 1, studies marked with an asterisk).

### 2.2. Task Selection

Our assessment of manuscripts found 59 eligible task-based neuroimaging studies, from which we selected tasks based on our integrative model of social cognition [32]. As described in the Introduction, the integrative model tied together different forms of social cognition by highlighting their overlap in the brain and proposing two overarching networks implicated across stimuli and tasks. From the sample of 59 eligible borderline personality disorder studies, we identified 19 studies linked to our model (see Table 1). Five studies featured emotion observation tasks [47,48,49,50,51]. Such tasks correspond to the “observing emotion” task type of our previous meta-analysis [32]. This kind of task can be considered a building block of empathy (e.g., [52,53]) or even a form of affect sharing (see, e.g., [54,55]). Three additional studies presented faces and asked for a mental state judgment [56,57,58], and therefore correspond to mentalizing tasks, such as the well-known Reading the Mind in the Eyes paradigm [59]. One task [60] presented an abstract–cognitive mentalizing task to patients, corresponding to the task group “trait judgments” from our previous meta-analysis [32]. Finally, ten tasks of the present literature sample featured a social interaction in the form of social exclusion (i.e., cyberball tasks [61,62,63,64]), social feedback [65,66,67], or an imagined social encounter [68,69,70]. Social interactions are considered a particularly ecologically valid way of measuring social cognition in the brain (see [43]), and neuroimaging studies found interaction-related brain activation to comprise multiple networks of our model of social cognition (see [32], pp. 20 and 23; see also [71]).

In addition to the 19 identified social tasks, we also found ten studies [72,73,74,75,76,77,78,79,80] that presented emotional (negative) pictures from the International Affective Picture System (IAPS, [81]) to participants. The IAPS features a variety of contents. Some images prominently feature facial expressions or social interactions and, therefore, are highly relevant for our meta-analysis. Other negative pictures are more non-social or physical, for example, depictions of dangerous animals, violence and death, or catastrophic events. For several studies, we could not find information regarding which IAPS pictures were selected for presentation and whether they showed socially relevant content (but see [75]). We therefore decided to carry out two meta-analyses, one time without and one time with IAPS studies. We will refer to these two instances as (i) meta-analyses for a narrow task selection and (ii) for an extended task selection (including IAPS tasks, *n* = 29, see Table 1 for details).

**Table 1:** Characteristics of included studies: Number of participants, description of tasks and stimuli.

Study	n1	n2	Task	Contrast
**Narrow task selection (*n* = 19)**				
Beeney [60]	17	21	Judging traits for self and other	Avg. activation for all judgments *
Bertsch [65]	48	28	Social Threat Aggression Paradigm	Aggressive > neutral cues in interaction
Cullen [47]	12	12	Viewing faces, implicit task	Covert fear > neutral faces
Guit.-M. [48]	10	10	Discriminating emotions/orientations	Fearful faces > neutral figures
Doell [66]	21	24	Monetary/social reward feedback task	Social feedback > non-social feedback
Domsalla [61]	20	20	Virtual ball-tossing game (cyberball)	Exclusion > obligatory inclusion
Fertuck [56]	16	17	Rating faces for trustworthiness or fear	Trustw.-to-untrustw. > fearful-to-neutral
Fertuck [62]	23	22	Virtual ball-tossing game (cyberball)	High > low rejection distress
Frick [57]	21	20	Reading Mind in the Eyes (RMET) task	Neg. > neutral emo. (affective mentalizing)
Goettlich [68]	19	22	Read scenarios and imagine taking part	Guilt scenarios (social content) > neutral
Herpertz [69]	33	30	Listen to script, imagine the scene	Avg. activation for interpersonal rejection *
Lamers [49]	20	20	Viewing movie sequences showing faces	Negative > neutral faces
Mier [58]	13	13	Judge intentions from emotional faces	Avg. activation for all judgments *
Nicol [50]	20	16	View faces and judge gender	Negative > neutral faces
Olie [63]	20	23	Virtual ball-tossing game (cyberball)	Exclusion > inclusion
Peters [70]	13	16	Directed Rumination Task	Content previous provocation > neutral
v. Schie [67]	26	32	Receiving feedback about an interview	Negative > positive feedback
Wrege [64]	39	29	Virtual ball-tossing game (cyberball)	Exclusion > inclusion
Wrege [51]	39	25	View faces and judge gender	Negative > neutral faces
**Additional tasks for extended task selection (combined *n* = 29)**				
Hazlett [72]	33	32	Judging valence of repeated IAPS pict.	Repeated unpleasant pictures *
Herpertz [73]	6	6	Passive viewing of IAPS pictures	Negative > neutral IAPS pictures
Koenigsb. [74]	18	16	Rating own emotion for IAPS pictures	Negative > neutral IAPS pictures
Koenigsb. [75]	19	17	Passive viewing of IAPS pictures	Negative IAPS > resting baseline *
Koenigsb. [82]	19	25	Viewing IAPS and Empathy ^1^ Pictures	Avg. activ. negative pictures *
Niedtfeld [76]	20	23	Passive viewing of IAPS pictures	Avg. activation for negative pictures *
Scherpiet [77]	18	18	Passive viewing of IAPS pictures	Negative > neutral IAPS pictures
Schnell [78]	14	14	Passive viewing of IAPS pictures	Avg. activation for negative pictures *
Schulze [79]	15	15	Passive viewing of IAPS pictures	Negative > neutral IAPS pictures
v. Zutph. [80]	55	42	Passive viewing of IAPS pictures	Negative > neutral IAPS pictures

* Marks studies that did not employ a high-level control condition. ^1^ Empathy Picture System (EPS, [83]). A more comprehensive version of this table is given in Supplementary Table S1.

### 2.3. Meta-Analysis Methods

We implemented a voxel-wise effect-size-based meta-analysis using Seed-based d Mapping with Permutation of Subject Images (SDM-PSI) software, version 6.21 ([44,84], www.sdmproject.com (accessed on 14 April 2024)). In brief (see [85]), we collected standard space coordinates and *t*- or *z*-values from study tables, based on which SDM-PSI estimates brain activity maps (voxel-wise effect sizes). Multiple imputations of the estimated effect sizes are employed to generate numerous image-based meta-analyses. The results of these analyses are then combined using Rubin’s rules. Note that SDM-PSI also implements measures to address potential methodological bias by adjusting for the sample size and the statistical threshold applied in the original analysis for each study. As a further measure to counteract risk of bias due to missing non-significant study results, SDM-PSI is capable of incorporating null findings (“no peaks” coordinate sets) in the analysis. Concretely, from the 29 studies included in our meta-analysis (see Table 1), 2 studies reported no significant group differences. For the present meta-analysis, we used SDM’s default preprocessing settings, employing a voxel size of 2 mm, smoothing with a full-width half maximum (FWHM) of 20 mm, and an anisotropic (α = 1.0) Gaussian kernel. We report results at a statistical voxel-level threshold of *p* < 0.05 family-wise error (FWE) corrected. For exploratory purposes, we additionally report results at an uncorrected voxel-level threshold of *p* < 0.005. We applied a minimum cluster extent of 10 voxels in all analyses. All results are reported in the MNI space. For completeness, we note that our meta-analyses were not preregistered.

We carried out two separate meta-analyses for the narrow and the extended task selection, each including the mean age and sex ratio of study participants averaged across patients and controls as covariates of no interest (see Supplementary Table S1 for details). In addition, we used SDM’s linear model function to contrast the narrow versus the extended task selection (i.e., non-IAPS versus IAPS tasks). Furthermore, we prepared a sub-group analysis for our narrow task selection meta-analysis, distinguishing between concrete and sensory-based versus abstract and transmodal stimuli. This distinction is based on [86], which identified a principal concrete-vs.-abstract gradient of functional brain organization. Our previous meta-analysis [32] found that this principal dimension of functional brain organization parsimoniously explains major aspects of brain activity patterns for social cognition.

We implemented additional analyses to assess our results’ robustness and potential bias. For the narrow task selection meta-analysis, we implemented a jack-knife sensitivity analysis (leave-one-out) to evaluate the robustness of results against the influence of individual studies. For the extended task selection, we implemented a subsampling approach to assess robustness and consider the difference in size between the narrow (*n* = 19) and the extended sample (*n* = 29). Across 100 repeats, we sampled 19 out of the 29 tasks in the extended selection, consisting of a fixed part containing all 10 IAPS tasks plus 9 additional tasks randomly drawn from the remaining studies. Based on this strategy, we estimated which results found for the extended task sample at full size (*n* = 29) would also be found for a smaller sample size comparable to that of the narrow task sample (*n* = 19). We also adapted the subsampling approach to match sample sizes in our linear model contrast analysis (*n* = 10 tasks from narrow selection vs. *n* = 10 IAPS tasks).

For the peak coordinates of our result maps, we computed heterogeneity statistics (I^2^ giving the relative amount of variance in study estimates attributable to heterogeneity rather than sampling error) and assessed publication bias with Egger’s test [87]. Following [10], we considered *p* values < 0.10 for Egger’s test to indicate publication bias.

## 3. Results

**Figure 2:**
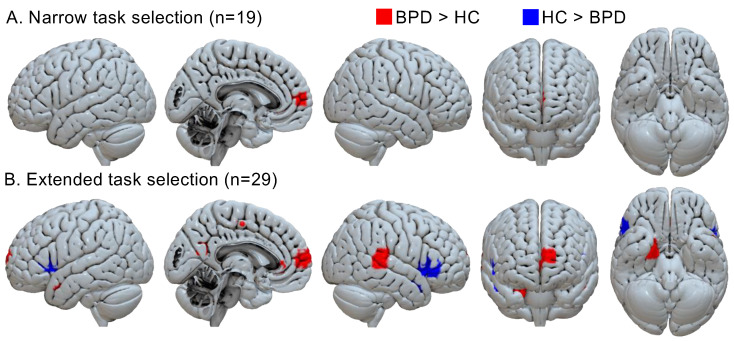
(**A**) Brain activation differences between patients with borderline personality disorder (BPD) and healthy controls (HCs) for the meta-analysis of our narrow selection of social cognition tasks (*n* = 19). Hyperactivations in patients with BPD are shown in red, and hypoactivations in blue. (**B**) Brain activation differences for our extended selection of social cognition tasks (including IAPS tasks, *n* = 29). All results are shown at an exploratory threshold of *p* < 0.005 uncorrected and minimum cluster size of 10 voxels.

We found no activations at a statistically corrected threshold of *p* < 0.05 (FWE corrected) for both meta-analyses using narrow and extended task selections. Figure 2 and Table 2 show the results we found for our exploratory threshold of *p* < 0.005 uncorrected and a minimum extent of 10 voxels. For the narrow task selection, we found hyperactivations for patients in the anterior cingulate and medial prefrontal cortex. We found no areas of reduced activation.

For our extended task selection (including IAPS tasks), we found the largest cluster of hyperactivation in the right parahippocampal gyrus and adjacent amygdala. Further clusters of hyperactivation were found in the right anterior cingulate cortex, medial prefrontal cortex (left superior frontal gyrus), right medial cingulate cortex, and right superior temporal gyrus (again see Figure 2 and Table 2). Additional smaller clusters of hyperactivation were found in the left precentral gyrus, left temporal pole, and left cuneus. In addition, we found functional hypoactivations for patients relative to controls in bilateral inferior frontal gyri and a small cluster in the right temporal pole. Table 2 (lower part) reports the percentage of permutation-based sub-samples of the extended task selection (i.e., reducing from *n* = 29 to *n* = 19) for which we found significant activation for the corresponding peak voxel at *p* < 0.005 uncorrected. Convergence for permutation-based sub-samples was highest for hyperactivations in the right amygdala (100/100 repeats) and right superior temporal gyrus (98/100), as well as for hypoactivations in the left inferior frontal gyrus (84/100).

A linear contrast analysis found no significant differences when comparing the narrow task selection versus the sample of IAPS tasks (*n* = 19 vs. *n* = 10). For the subsampling approach matching sample sizes (*n* = 10 narrow task selection vs. *n* = 10 IAPS tasks), initial results across 50 repeats found no activation differences (i.e., differences were found in 0/50 repeats). Moreover, we did not run our additionally planned linear contrast analysis comparing abstract versus concrete tasks, as our coding of studies (see Table 1) revealed a large imbalance and too few studies for the abstract category (*n* = 14 concrete, *n* = 5 abstract tasks).

We also assessed publication bias for all cluster peaks of the narrow task selection meta-analysis and for all peaks of clusters of >20 voxels for the extended task selection. Egger’s test [87] indicated no significant publication bias for any of the peak coordinates (see Supplementary Table S2 for details).

## 4. Discussion

The present study aimed to clarify the neural bases of impaired social cognition in borderline personality disorder. As neuroimaging studies on borderline personality disorder have been increasing in numbers, we sought the opportunity to meta-analyze consistencies in neural abnormalities across a subset of tasks and experiments involving social cognition. Our study builds on previous meta-analyses, which explored brain abnormalities in borderline personality disorder across all tasks [9,10] or selectively studied emotion-processing tasks [8,9,10,88]. In contrast to these previous meta-analyses, we sought to distinguish tasks focusing on emotion processing from those involving social cognition. Therefore, we filtered borderline personality studies based on the task used, following the model of social cognition we have proposed previously [32]. Concretely, we included emotion observation tasks (faces), mental state judgment tasks, social exclusion tasks, and social interaction tasks. To select all social-cognition-related tasks, we ultimately carried out two meta-analyses using a narrow and an extended task sample. The difference between those samples concerns the inclusion of tasks presenting stimuli from the International Affective Picture System (IAPS). The IAPS contains diverse images; some images prominently feature facial expressions or social interactions and, therefore, are highly relevant for our meta-analysis. Other negative pictures are more non-social or physical, for example, depictions of dangerous animals, violence and death, or catastrophic events. Not all studies provided details on selecting IAPS pictures presented to participants, so we only included these stimuli in our extended task selection.

### 4.1. Meta-Analysis of the Extended Task Selection

Our extended social task sample (including IAPS tasks) partially converges with task samples from previous meta-analyses on emotion processing in BPD [8,9,10]. However, while many of the studies in our samples feature emotional content, the tasks we selected had to engage participants in social cognition (as defined by our previous meta-analysis [32]). This implies that we removed tasks featuring unrelated processes from our analysis. Concretely, we removed cognitive tasks featuring emotional distractors (e.g., flanker tasks) and tasks embedding emotional stimuli in cognitive tasks (e.g., emotional working memory). In addition, we removed tasks presenting emotion words in the present meta-analysis since single words are not related to a specific person (self or other). This task selection strategy differs from several previous meta-analyses [8,9,88], which aimed to capture a broad range of emotion-processing tasks for a robust summary.

The results of our extended task selection meta-analysis show limited overlap with previous findings. At an exploratory threshold of *p* < 0.005 uncorrected, we found the largest cluster of hyperactivation for patients with BPD in the right parahippocampal gyrus and adjacent amygdala. In proximity to our peak coordinates, activation was also found in [9,10] (proximity denotes a Euclidean distance <20 mm, which corresponds to the smoothing kernel of our meta-analysis). We did not find activation in the left amygdala but in a small cluster of the left temporal pole (although anatomically distinct, Euclidean distance was <20 mm compared to the left amygdala coordinates of [8,9]). We further found hyperactivation in medial prefrontal areas, partially overlapping with findings of other meta-analyses. Specifically, [8] reported activation in proximity to our vmPFC peak (MNI coordinates x = −8, y = 60, z = 12), and [10] reported activation near our ACC peak (MNI coordinates x = 12, y = 44, z = 4). Further correspondences regarding nearby peak coordinates were found with [10] for the right superior temporal gyrus and [8] for the left precentral gyrus and cuneus. In terms of hypoactivations for patients relative to controls, our meta-analysis mainly found activation differences in bilateral inferior frontal gyri. This finding was only partially reflected by one other meta-analysis [8], where activation coordinates were reported in the left precentral gyrus and right insula (at a more substantial distance compared to our peaks; Euclidean distances were between 20 and 30 mm).

Taken together, the comparison between meta-analyses identifies the right amygdala and vmPFC/ACC as the most convergent loci of hyperactivation and bilateral inferior frontal areas as potential loci of hypoactivation (although less support is found for the latter areas). This pattern of findings has also been mentioned in previous meta-analyses [8,9] and literature reviews [6,7,15,89]. Several authors have linked prefrontal–limbic abnormalities to emotion dysregulation in BPD [3,6,7,90]. For example, Linehan’s biosocial developmental model assumes that BPD is primarily a disorder of emotion dysregulation, which is based on a heritable component that becomes amplified by environmental influences [3,90]. Relatedly, in our previous studies [15,17] and related work [89], we and others have proposed specific functional roles of the amygdala and vmPFC, as well as bilateral inferior frontal gyri, in emotion processing in BPD (and how these processes could be implicated in social interactions). Specifically, previous works [15,17,89] have linked functional interpretations of the amygdala and vmPFC in terms of encoding the expected valence of stimuli (see, e.g., [91,92]) to the rigid interpretation of interpersonal situations in BPD and resulting impulsive behavior. For the left inferior frontal gyrus, we [93] and others [94] have discussed potential roles for controlled semantic elaboration. While the meta-analysis of the extended task selection largely supports these previous interpretations, the present study sought to work out a novel and distinct research question, namely specifying the neural correlates of aberrant social cognition in BPD.

### 4.2. Meta-Analysis of the Narrow Task Selection

Our narrow task selection is of central interest for our study’s aim to specify brain abnormalities for social cognition in borderline personality disorder. Note that another recent study [10] also carried out a meta-analysis segregating different task types (as a supplementary analysis), sorting out both emotion tasks and social cognition tasks. As reviewed in the previous section, we found overlapping activation in the right amygdala between this emotion task meta-analysis [10] and our extended task selection meta-analysis. Due to sample size limitations and a different research question (comparing brain activation for BPD versus ADHD), that study [10] did not carry out a separate meta-analysis for social cognition tasks in patients with BPD (see [10] Supplementary Materials p. 60).

In the present meta-analysis, we laid a focus on social cognition tasks in BPD by applying a narrow (i.e., strict) task selection strategy to the literature. For this meta-analytic approach, we only found group differences in terms of hyperactivation for patients with borderline personality disorder. All three activation clusters found were located in the medial prefrontal cortex (superior frontal gyrus, BA 10) and adjacent anterior cingulate cortex (BA 32). Mapping the peak coordinates of these three clusters (see Table 2, upper section) based on an atlas of resting-state fMRI networks [95] showed that all were located in the default mode network. To further characterize the medial prefrontal hyperactivations we found, we compared the location of peak coordinates to overarching social cognition brain networks in healthy adults, which we found in a meta-analysis [32]. The main result of our previous work was generated by meta-analytic clustering, showing three main patterns of brain activation for social cognition. One distinctive pattern (cluster 1, “cognitive processes”) was centered on areas of the default mode network and linked to social cognition tasks, which involve forming more abstract and decoupled representations of others’ mental states. Another individual pattern (cluster 3, “affective processes”) comprised areas of the ventral attention (salience) and somatosensory networks and was linked to tasks involving shared emotional, motor, and somatosensory representations of others’ affective states. Finally, we found several social cognition tasks that consistently co-recruit cognitive and affective processes (cluster 2, “combined processes”), i.e., a pattern of activation combining parts of clusters 1 and 3. Comparing the three overarching networks characterizing social cognition to our results, we found that all hyperactivations found for patients with BPD (medial prefrontal and anterior cingulate) were localized within the cognitive processing network (cluster 1). In [32], we discussed stimuli and tasks that were found to activate the cognitive processing network in healthy adults and extended this perspective with neurosynth functional decoding [96]. Concerning social topics, neurosynth decoding found the strongest associations with the terms “theory of mind”, “mentalizing”, and related concepts. For non-social topics, we found the strongest associations with “default”, “self-referential”, and “autobiographical”. These results supported functional interpretations in terms of self-generated cognition [97], emphasizing the role of the default mode network for understanding others (see [29,32,98,99,100,101,102,103]). This functional perspective on medial prefrontal/anterior cingulate hyperactivations in borderline personality disorder can be reconciled with previous research (e.g., [104,105]). In clinical and behavioral assessments, patients with BPD show impairments in the ability to correctly infer the mental states of other people (for review, see [19,20,21]). Previous work suggests [21,24,26] that patients with BPD strongly rely on fast and automatic forms of mentalizing in combination with problems in controlled and cognitive mentalizing. Concerning this imbalance in processes for understanding others, it was noted that patients with BPD tend to conflate mental states of the self and others [21] and are impaired in drawing the “self–other distinction” (e.g., shifting between self and other representations according to task demands; see [19]). In light of these deficits, the medial prefrontal/anterior cingulate hyperactivations observed in our meta-analysis could signify an overreliance on self-projection in patients with BPD. According to an extensive meta-analysis of neuroimaging studies in healthy adults [28], medial prefrontal areas implicated in social cognition can be divided into dorsal versus ventral aspects, with an approximate boundary between the parts at z = 20. Correspondingly, the peak coordinates of hyperactivations found in our meta-analysis fell in the ventral part of the medial prefrontal/anterior cingulate areas, with z-axis coordinates of 0, +8, and +10 for the three cluster peaks (see Table 2, upper section). Several studies have pointed out a prominent role of the ventromedial prefrontal cortex in self-projection, using knowledge about one’s own thoughts, feelings, and preferences as a guide to understanding others [106,107,108]. For example, studies found that brain activation in the ventral (but not dorsal) mPFC was higher for mentalizing about similar compared to dissimilar others [107,109] and that the agreement between one’s own choices and the predicted choices for other people was stronger when activation in the ventral mPFC was high during prediction [108]. In contrast, studies focusing on the ability to successfully draw a “self–other distinction” found that the temporo-parietal junction—rather than the medial prefrontal cortex—plays a central role in promoting this capacity (see, e.g., [19,110,111,112]). Taken together, these findings on the functional role of vmFPC suggest that BPD hyperactivations may reflect an overreliance on self-related thought to understand the mental states of others.

From a brain network perspective, previous functional accounts (e.g., [8,15,89]) considering the vmPFC’s role in BPD beyond the social context are broadly consistent with the present interpretation. Correspondingly, previous accounts are aligned with the default mode network’s overarching role in mediating self-referential cognition and autobiographical thought. A previous meta-analysis of negative emotion-processing studies in BPD [8] interpreted observed hyperactivations in default mode areas in terms of an increased self-related interpretation of negative stimuli that may trigger more ruminative thoughts and autobiographical content. Moreover, theoretical models of change in psychotherapy [15] and of emotion regulation [89] discuss related concepts. These models assume that semantic representations that are based on autobiographic experiences are foundational for the understanding of the self, others, and relationships between past experiences and current interpersonal situations. These representations function as schemas or mental structures that organize and interpret the emotional significance of our daily experiences. With respect to social cognition and interpersonal relationships, attachment theory highlights an important role of early caregiver experiences as an important source for inner working models, i.e., autobiographically mediated mental schemas (see, e.g., [14,113,114,115,116,117]). This notion offers a theoretical link between the presently found hyperactivation in vmPFC for patients with BPD (linked to increased self-related thought and autobiographical representations) and the prevalence of adverse attachment experiences, especially relationship traumas related to maltreatment and physical abuse in patients with BPD (e.g., [14,16,17,117,118,119,120].

### 4.3. The Relation between Results of the Extended and Narrow Task Selection

Despite our social-cognition-related interpretation of vmPFC hyperactivations in patients with BPD, we found similar group differences for our narrow and extended task selection meta-analyses in the medial prefrontal cortex and anterior cingulate cortex. Notably, the direct comparison of activation between task selections did not reveal any significant differences. The conceptual difference between the narrow and extended task samples was the inclusion of IAPS tasks. As we have argued, some IAPS stimuli feature social content, but other pictures of the set might be socially less relevant. Therefore, the fact that the extended task selection activated the same areas as the narrow task selection does not provide conclusive evidence.

### 4.4. Limitations

In addition to the mentioned limitation regarding the comparison of narrow versus extended task selections, our meta-analysis shows several other limitations. First, the literature review for this meta-analysis found that most studies featured social and emotional content conjointly (also in our narrow task selection). Arguably, such stimuli are ecologically highly relevant. However, they also limit the potential of our meta-analysis to segregate purely cognitive from more intermediate (cognitive and affective combined) forms of social cognition. Second, similar to previous meta-analyses (e.g., [9]), we observed limited convergence regarding the neurofunctional correlates of BPD. That is, we found no significant group differences in brain activation for either the narrow or the extended task selection at a statistically corrected threshold (FWE, *p* < 0.05). We discuss in this manuscript results for an exploratory threshold of *p* < 0.005 uncorrected, which must be understood as preliminary evidence. One likely factor contributing to the limited convergence in brain activation is the inclusion of studies containing patients currently taking medication and showing multiple diagnoses. Concerning concurrent medication, a previous meta-analysis [9] of emotion-processing tasks in BPD found similar results for a sub-sample of only unmedicated patients and a larger sample of medicated and unmedicated patients. Neuroimaging studies of BPD often feature samples with marked comorbidity (for an overview, see, e.g., [9,10]), with frequent concurrent diagnoses being, for example, PTSD [121] and depression [122]. Our meta-analysis only considered the comparison of patients with BPD versus healthy controls. However, a previous meta-analysis [8] showed that some brain abnormalities found in patients with BPD are also found in patients with major depression and PTSD. These findings highlight the interpretational limitations of our results, which may not be linked to BPD alone. A third limitation of our study is that we omitted the planned comparison between tasks showing concrete social stimuli (i.e., pictures or drawings of faces, persons, or interactions) and abstract tasks (i.e., purely verbal). We intended to carry out this comparison to follow up on the observation from our previous meta-analysis [32] that brain activation patterns across different social cognition tasks largely reflect a principal concrete-vs.-abstract gradient of functional brain organization (see [86]). However, our coding of tasks into corresponding categories produced a significant imbalance in sample size (*n* = 14 concrete, *n* = 5 abstract tasks), and we therefore did not compare the two types of tasks. Based on the limitations of our meta-analysis and our review of studies, we conclude that additional evidence is required to delineate current sources of heterogeneity. Future neuroimaging studies presenting both emotional and social tasks to a larger sample of patients with BPD could help differentiate heterogeneity in brain activation related to the type of task, medication status, and comorbid diagnoses.

## 5. Conclusions

Our meta-analysis found a frequent co-occurrence of social and emotional contents in studies probing the neural correlates of borderline personality disorder. When applying a more liberal strategy for task selection to that literature (including social and emotional contents), our results partially converge with previous findings and identify the right amygdala and parts of the medial prefrontal cortex as the primary loci of hyperactivation in BPD. When testing for activation differences in the opposite direction, we found hypoactivations in bilateral inferior frontal gyri for patients. A second meta-analysis, employing a stricter approach to only select social cognition tasks, found hyperactivations only in prefrontal areas, particularly in the ventromedial prefrontal cortex (which was also activated in the extended meta-analysis). Based on evidence of the vmPFC’s role in social cognition in healthy individuals, we suggest that BPD hyperactivations in the area reflect an overreliance on self-related thought to understand the mental states of others. This observation could reflect a neural underpinning of BPD patients’ difficulties with mentalizing, in particular, the tendency to conflate mental states of the self and others [21].

## Figures and Tables

**Table 2:** Locations of activation for narrow and extended task selection meta-analyses.

	Cluster Peak						Sub-Peaks			
Label	x	y	z	z-Val	jk/Perm.	vx	x	y	z	Label
*Narrow task selection (n = 19)*										
Borderline personality disorder > healthy controls										
R ant. cing. g.	8	38	0	3.10	17/19	30	12	46	2	R ant. cing. g.
L sup. front. g.	−8	58	10	3.23	17/19	13				
L ant. cing. g.	−12	46	8	3.31	17/19	12				
Healthy controls > borderline personality disorder										
*Extended task selection (including IAPS tasks, n = 29)*										
Borderline personality disorder > healthy controls										
R parahipp. g.	22	0	−26	3.43	100	246	26	−4	−24	R parahipp. g.
							22	−3	−16	R amygdala
R ant. cing. g.	12	44	4	3.94	29	118	10	38	−2	R ant. cing. g.
R sup. temp. g.	64	−32	12	3.25	98	93	64	−38	4	R mid. temp. g.
L sup. front. g.	−8	60	12	3.75	28	72				
R med. cing. g.	8	−16	50	3.41	78	44				
L prec. g.	−42	2	28	3.03	8	16				
L cuneus	−8	−62	22	2.78	21	14				
L temp. pole	−46	4	−16	2.74	31	14				
Healthy controls > borderline personality disorder										
R inf. front. g.	46	18	2	3.55	69	212	42	24	−2	R insula
							50	30	2	R inf. front. g.
L inf. front. g.	−48	20	4	3.25	84	60	−40	18	0	L insula
R temp. pole	48	6	−22	2.92	31	16				

Results are reported at an exploratory threshold of *p* < 0.005 uncorrected and a minimum cluster size of 10 voxels. jk gives the number of leave-one-out jack-knife repeats (out of 19) for which we found significant activation at the given peak voxel for the exploratory statistical threshold. Perm. gives the percentage (i.e., *n* out of 100) of permutation-based sub-samples reducing the extended task selection to *n* = 19 (keeping all IAPS tasks), for which we found significant activation at the given peak voxel for the exploratory statistical threshold.

## Data Availability

No new data were created in this meta-analysis. Coordinate files and result maps from the meta-analysis are available upon request.

## Supplementary Materials

The following supporting information can be downloaded at: https://www.mdpi.com/article/10.3390/brainsci14040395/s1, Table S1: Characteristics of included studies; Table S2: Heterogeneity statistics and Egger’s test.
