# Supplementary material for: Brain Activation for Social Cognition and Emotion Processing Tasks in Borderline Personality Disorder: A Meta-Analysis of Neuroimaging Studies
Source: Brain Sci. 2024 Apr 18;14(4):395. doi: 10.3390/brainsci14040395 (PMC11048542; doi:10.3390/brainsci14040395)
Supplement: Supplementary file 1 [file brainsci-14-00395-s001.zip › Supplementary_Table_S1.pdf]

Supplementary Table S1

Characteristics of included studies: Number of participants, mean age, sex ratio, medication status, description of tasks and stimuli.

Study	BPD				Controls			Task	Contrast	
	N	Age	N male	N med	N	Age	N male			
Narrow tasks selection (n=19)										*Abstract Stim.*
Beeney 2016	17	35.51	0	10	21	33.33	0	Judging personality traits for self and other	Avg. activation for all judgments*	+
Bertsch 2022	48	29.6	0	0	28	27.8	0	Social Threat Aggression Paradigm	Aggressive cues > neutral cues in interaction	
Cullen 2016	12	25.2	0	1	12	24.2	0	Viewing faces, covert presentation, implicit task	Covert fear > neutral faces	
Doell 2020	21	27.43	0	13+	24	24.71	0	Monetary and social reward feedback task	Social feedback > non-social feedback	
Domsalla 2014	20	29.20	0	0	20	28.70	0	Virtuall ball tossing game (cyberball)	Exclusion > Obligatory inclusion	
Fertuck 2019	16	25.94	0	0	17	23.71	0	Rating faces regarding trustworthiness or fear	Trustworthy-to-untrustw. > fearful-to-neutral	
Fertuck 2023	23	26.26	0	0	22	25.95	0	Virtuall ball tossing game (cyberball)	High > low rejection distress during exclusion	
Frick 2012	21	27.14	0	0	20	24.80	0	Reading Mind in the Eyes (RMET) task	Neg. > neutral emotion (affective mentalizing)	
Goettlich 2020	19	26.50	0	9+	22	26.40	0	Read scenarios and imagine being part	Guilt scenarios (social content) > neutral	+
Guitart-M. 2009	10	31.3	5	0	10	31.2	5	Discrimination of face emotions / figure orientations	Fearful faces > neutral figures	
Herpertz 2017	33	26.19	0	0	30	27.69	0	Listen to script, imagine the scene	Avg. activation for interpersonal rejection phase*	+
Lamers 2019	20	25.95	0	0	20	26.90	0	Viewing movie sequences showing faces	Negative > neutral faces	
Mier 2013	13	28.15	4	12	13	30.46	5	Judge intentions from emotional faces	Avg. activation for all judgments*	
Nicol 2015	20	35.6	3	15+	16	35.7	2	View faces and judge gender	Negative > neutral faces	
Olie 2018	20	26.95	0	12+	23	25.00	0	Virtuall ball tossing game (cyberball)	Exclusion > inclusion	
Peters 2018	13	21.81	0	2	16	21.23	0	Directed Rumination Task	Content is previous provocation > is neutral	+
van Schie 2020	26	30.46	0	3	32	28.12	0	Receiving feedback about an interview	Negative (e.g. "arrogant") > positive feedback	+
Wrege 2019	39	27.50	9	23	29	25.70	4	Virtuall ball tossing game (cyberball)	Exclusion > inclusion	
Wrege 2021	39	28.4	10	24	25	25.8	4	View faces and judge gender	Negative > neutral faces	
Additional tasks for extended task selection (combined n=29)										
Hazlett 2012	33	31.6	0	0	32	32.8	0	Judging valence of IAPS pictures, repeat. present.	Repeated unpleasant pictures*	
Herpertz 2001	6	26.2	0	0	6	27.2	0	Passive viewing IAPS pictures	Negative > neutral IAPS pictures	
Koenigsb. 2009a	18	32.6	8	0	16	31.8	7	Viewing IAPS pictures, rating own emotional react.	Negative > neutral IAPS pictures	
Koenigsb. 2009b	19	34.9	12	0	17	31.2	9	Passive viewing IAPS pictures	Negative IAPS > resting baseline*	

Koenigsberg 2014	19	31.9	8	0	25	28.1	12	Viewing pictures from IAPS and Empathy Pict. Sys.	Avg. activation negative pict. (novel + repeated)*
Niedtfeld 2010	20	30.5	0	0	23	27.1	0	Passive viewing IAPS pictures	Avg. activation for negative pictures*
Scherpiet 2014	18	28.4	0	11	18	28.9	0	Passive viewing IAPS pictures	Negative > neutral IAPS pictures
Schnell 2007a	14	28.0	0	0	14	28.4	0	Passive viewing IAPS pictures	Avg. activation for negative pictures*
Schulze 2011	15	27.6	0	0	15	24.5	0	Passive viewing IAPS pictures	Negative > neutral IAPS pictures
van Zutphen 2018	55	30.8	0	38	42	28.3	0	Passive viewing IAPS pictures	Negative > neutral IAPS pictures

N med ... reports the number of patients who were medicated at the time of the scan; n+ indicates multiple medications involving at least n patients.* ... marks studies which did not employ a high-level control condition. + ... marks abstract (i.e., purely verbal) tasks, all other tasks showed concrete social stimuli (i.e., pictures or drawings of faces, persons, or interactions).
