# Supplementary material for: Brain Activation for Social Cognition and Emotion Processing Tasks in Borderline Personality Disorder: A Meta-Analysis of Neuroimaging Studies
Source: Brain Sci. 2024 Apr 18;14(4):395. doi: 10.3390/brainsci14040395 (PMC11048542; doi:10.3390/brainsci14040395)
Supplement: Supplementary file 1 [file brainsci-14-00395-s001.zip › Supplementary_Table_S2.pdf]

Supplementary Table S2. Heterogeneity statistics I^2, and results of Egger's tests for cluster peaks from narrow and extended task selection meta-analyses.

	mni				Egger's test			
Label	x	y	z	I^2	Bias	z	df	p
Narrow task selection								
Borderline personality disorder > healthy controls								
R ant. cing. g.	8	38	0	14.399345	0.05	0.03	17	0.975
L sup. front. g.	-8	58	10	4.218039	0.03	0.02	17	0.983
L ant. cing. g.	-12	46	8	5.880384	0.02	0.01	17	0.99
Extended task selection (only clusters > 20 voxels)								
Borderline personality disorder > healthy controls								
R parahipp. g.	22	0	-26	8.253916	-0.01	-0.01	27	0.994
R ant. cing. g.	12	44	4	11.747099	0	0	27	1
R sup. temp. g.	64	-32	12	0.834312	0.01	0.01	27	0.988
L sup. front. g.	-8	60	12	1.860792	-0.01	-0.01	27	0.995
R med. cing. g.	8	-16	50	12.574807	0.01	0.01	27	0.991
Healthy controls > borderline personality disorder								
R inf. front. g.	46	18	2	9.62053	0.02	0.02	27	0.985
L inf. front. g.	-48	20	4	3.499163	-0.01	-0.01	27	0.992
